# The Golgi complex in stress and death

**DOI:** 10.3389/fnins.2015.00421

**Published:** 2015-11-06

**Authors:** Carolyn E. Machamer

**Affiliations:** Department of Cell Biology, Johns Hopkins University School of MedicineBaltimore, MD, USA

**Keywords:** Golgi complex, disassembly, stress, apoptosis, signaling

## Abstract

The Golgi complex is a central organelle of the secretory pathway where sorting and processing of cargo occurs. While Golgi structure is important for the efficient processing of secretory cargo, the unusual organization suggests additional potential functions. The Golgi is disassembled after various cellular stresses, and we hypothesize that Golgi disassembly activates a stress signaling pathway. This pathway would function to correct the stress if possible, with irreparable stress resulting in apoptosis. Neurons appear to be particularly sensitive to Golgi stress; early disassembly of the organelle correlates with many neurodegenerative diseases. Here, Golgi stress and potential signaling pathways to the nucleus are reviewed.

## Golgi structure and function

The Golgi complex plays a central role in processing and sorting of biosynthetic cargo in all eukaryotic cells. In mammals, the Golgi complex consists of sets of flattened cisternal membranes arranged in stacks with associated tubules and vesicles, which are usually collected at the microtubule organizing center (MTOC) in a ribbon structure (Klumperman, 2011). This structure is not essential for the known functions of the Golgi, and may suggest additional functions. Golgi structure is also quite dynamic; the organelle is disassembled at mitosis and then reassembled (Wang and Seemann, 2011). The organelle can also accommodate cargo of different shapes and sizes (Machamer, 2013). We previously hypothesized that mammalian Golgi organization may have evolved in part to sense and transduce specific stress signals to the nucleus (Hicks and Machamer, 2005).

**Figure 1:**
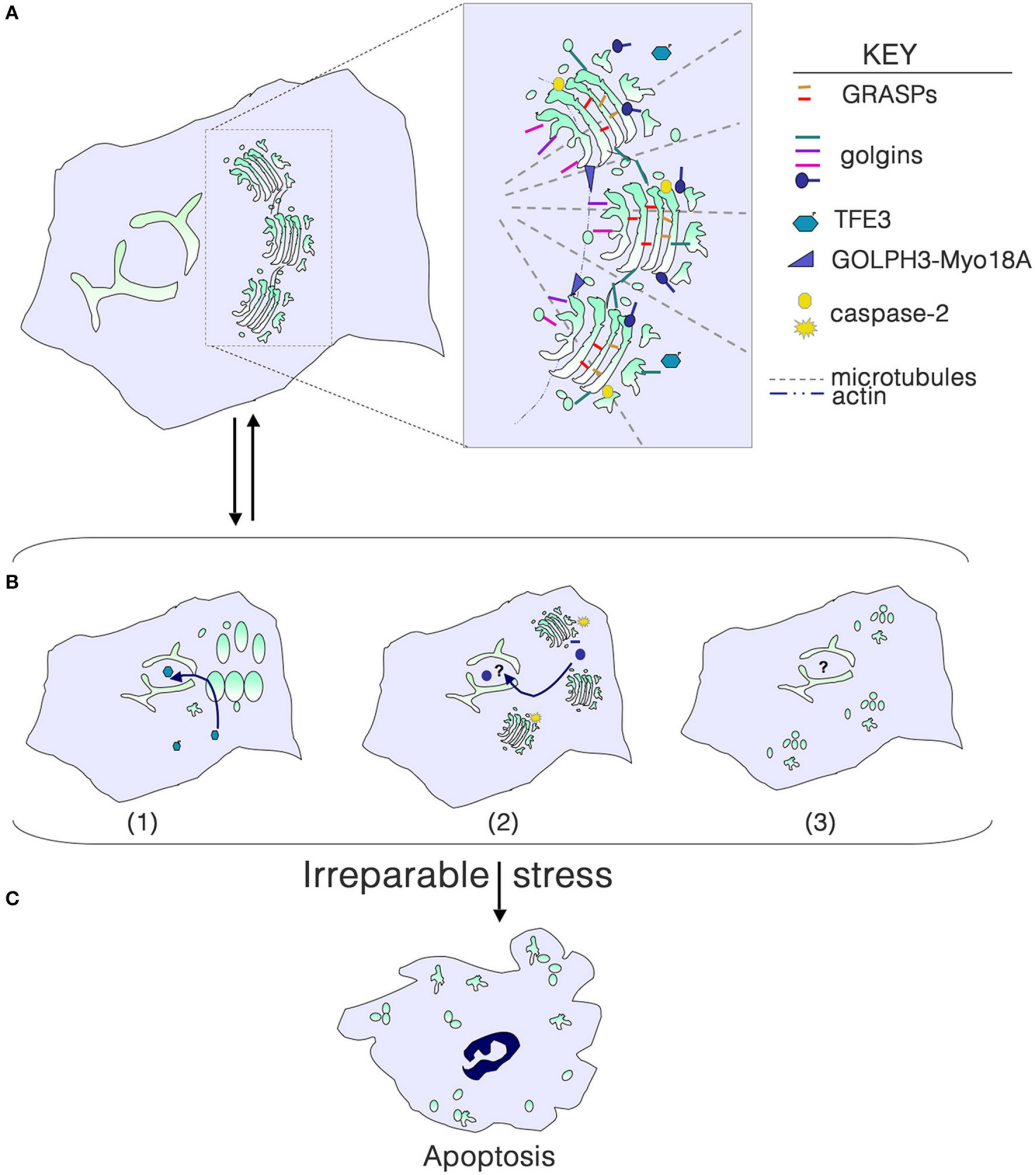
**Golgi structure in life, stress and death**. **(A)** Golgi morphology in a typical mammalian cell, with the key structural players shown in the inset. For simplicity, individual golgins and GRASPs are not indicated. **(B)** Golgi stress due to cargo load or size, ionic imbalance, infection with intracellular pathogens, or perturbation of glycosylation or the cytoskeleton results in structural alterations that can signal to the nucleus to help repair the stress. (1) Dephosphorylation of TFE3 and tranlocation to the nucleus results in transcription of genes with a GASE, including some glycosyltransferases and trafficking components. (2) Activation of local caspase-2 cleaves select golgins, and fragments enter the nucleus to perform an unknown function. (3) Phosphorylation of GRASPs and golgins or their cleavage can result in a more complete disassembly of the Golgi, although the consequences for signaling to the nucleus are unknown. **(C)** With irreparable stress, the Golgi is completely disassembled as the cell undergoes apoptosis.

Golgi structure in mammalian cells is maintained by the cytoskeleton, and GRASPs and golgins (Figure 1A). GRASP65 and GRASP55 form homo- or hetero-oligomers and mediate stacking and can contribute to the Golgi ribbon structure (Ramirez and Lowe, 2009; Xiang and Wang, 2010). The golgin family comprises a group of peripheral Golgi membrane proteins with long coiled coil domains. Some golgins are vesicle tethers, some function in Golgi stack structure, and others may be involved in trafficking of specific cargo molecules (Munro, 2011). Disassembly of the Golgi in mitosis or apoptosis results from reversible phosphorylation of GRASPs and golgins or irreversible cleavage, respectively.

## Golgi disassembly and stress

Golgi fragmentation is commonly observed in cells subjected to “stress,” including pharmacological and oxidative stress. Fragmentation can be the result of perturbation of microtubules, or phosphorylation or cleavage of Golgi structural proteins. Golgi stacks can be dispersed (mini-stacks) or completely disassembled, depending on the perturbation (Figure 1B). Although the term “Golgi stress” has been frequently used in the literature (e.g., Jiang et al., 2011; Oku et al., 2011; Reiling et al., 2013), there is no clear understanding of what Golgi stress entails. Can Golgi stress be activated in the absence of endoplasmic reticulum (ER) stress? Similar to the well-documented unfolded protein response in the ER (Walter and Ron, 2011), a Golgi stress response pathway should serve to help alleviate the stress, and only result in cell death if the stress is irreparable (Figure 1C). Pharmacological inhibitors of glycosyltransferases, glycosidases, proton and calcium pumps, and perturbation of luminal pH have all been shown to alter the structure of the Golgi complex. High levels of cargo or large cargo passing through the Golgi may be the most physiological type of stress. But do any of these insults result in outcomes that would help eliminate the stress?

One of the most extensively studied types of cellular stress is pro-apoptotic stress. In apoptosis, extrinsic, or intrinsic pathways lead to programmed disassembly of the cell. Cysteine proteases called caspases are activated and cleave a select set of cellular proteins during programmed cell death. Different types of stress activate specific initiator caspases, which then activate effector caspases (Boatright and Salvesen, 2003). Not all caspases are involved in cell death however.

We previously reported that procaspase-2 is partially localized at the cytoplasmic face of the Golgi complex (Mancini et al., 2000), and golgin-160 and several other golgins are caspase-2 substrates (Mancini et al., 2000; Lowe et al., 2004). Caspase cleavage of golgin-160 is predicted to inhibit its function in promoting efficient trafficking of specific cargo molecules (Bundis et al., 2006; Hicks et al., 2006; Williams et al., 2006). Caspase-2 is an unusual caspase in that it possesses a long prodomain like inititator caspases, but does not activate effector caspases (Fava et al., 2012). Recent evidence suggests non-apoptotic roles for caspase-2 in maintaining genome stability, checkpoint regulation in the cell cycle, response to oxidative stress, tumor suppression and in aging (Olsson et al., 2015).

Another stress that acts at the Golgi complex is inhibition of O-glycosylation. It was shown that treatment of fibroblasts with benzyl 2-acetamido-2-deoxy-a-d-galactopyranoside (GalNAc-bn) to inhibit this post-translational modification induced upregulation of HSP47, an ER chaperone (Miyata et al., 2013). HSP47 apparently protects cells from Golgi fragmentation and death when O-glycosylation is blocked because knock-down of HSP47 in GalNAc-bn treated cells led to Golgi vacuolization and eventual apoptosis. Interestingly, caspase-2 appears to be activated here as well. The mechanism by which HSP47 leads to protection is unknown, but it has been reported to be a collagen-specific chaperone (Mala and Rose, 2010). HSP47 may regulate the level of this abundant secretory protein that enters the Golgi, whereas in its absence collagen might accumulate in the Golgi when it cannot be O-glycosylated, resulting in Golgi structural perturbations and eventual apoptosis.

## Golgi-nucleus signaling pathways

Signals from the nucleus can lead to phosphorylation of Golgi proteins and subsequent Golgi disassembly. For example, GOLPH3 is an oncogene that is upregulated in a number of cancers. It is a peripheral Golgi protein that binds phosphoinositol-4-phosphate at the *trans*-Golgi as well as the myosin Myo18A (Dippold et al., 2009). Normal Golgi ribbon structure is maintained in part by interaction of the Myo18A-GOLPH3 complex with actin, which keeps the ribbon extended. DNA damage results in Golgi fragmentation, which was shown to occur through phosphorylation of GOLPH3 by DNA-dependent protein kinase (DNA-PK). Phosphorylated GOLPH3 interacts more strongly with Myo18A and thus leads to Golgi dispersal (Farber-Katz et al., 2014). Although this dispersal leads to a reduction in cargo traffic through the Golgi, it is not yet clear how this connects to DNA damage or cell survival. Since DNA-PK can activate caspase-2 (Shi et al., 2009), it will be important to determine if caspase-2 cleavage of Golgi proteins contributes to this response.

But can signals from the Golgi lead to changes in gene expression in the nucleus? Membrane-anchored transcription factors can be released from Golgi membranes by site 1 and 2 proteases (Fox and Andrew, 2015), allowing nuclear translocation and transcription of specific genes. However, these transcription factors are trafficked to the Golgi by specific events signaled in the ER. Several golgins contain cryptic nuclear localization signals, and fragments of these proteins (generated by caspases or other proteases) can be targeted to the nucleus. But to date, none of these fragments has been shown to induce gene expression that might alter Golgi function. A caspase cleavage fragment of p115, a vesicle tethering protein localized at the *cis*-Golgi, is targeted to the nucleus and promotes cell death in a p53-dependent pathway (Chiu et al., 2002; How and Shields, 2011). However, cleavage of p115 is not observed in all pro-apoptotic settings (Lowe et al., 2004).

A cryptic nuclear localization signal is present in a fragment of golgin-160 generated by caspase-2 cleavage, and this fragment accumulates in the nucleus when expressed exogenously (Hicks and Machamer, 2002). We hypothesized that Golgi stress leads to low levels of caspase-2 activation, and that the nuclear fragments of golgin-160 participate in a stress repair pathway (Hicks and Machamer, 2005). The nuclear fragments do not resemble transcription factors *per se*, so may serve as transcriptional enhancers or repressors. Interestingly, cells expressing caspase-resistant golgin-160 were less sensitive to apoptosis induced by ER stress and death receptor ligation compared to cells expressing wild-type golgin-160. However, these cells responded normally to other pro-apoptotic stresses (Maag et al., 2005). Our recent data support the idea that the stable lines expressing caspase-resistant golgin-160 adapted during selection because a block in golgin-160 cleavage prevented normal response to stress. Although it is interesting that only stress within the secretory pathway depends on golgin-160 cleavage, we still lack direct evidence that golgin-160 is cleaved during Golgi stress and mediates subsequent changes in gene expression.

Extended treatment with the ionophore monensin was shown to increase the levels of certain Golgi glycosylation enzymes and vesicle transport components (Oku et al., 2011). Monensin collapses sodium and proton gradients, and thus raises the pH and induces swelling of low pH compartments, including the Golgi complex. The authors identified a “Golgi apparatus stress element” (GASE) in the promoters of several Golgi resident proteins that was required for their upregulation. Further work identified TFE3, a basic helix-loop-helix transcription factor, which binds to the GASE (Taniguchi et al., 2015). They showed that under normal growth conditions, TFE3 was phosphorylated and remained cytoplasmic, but after monensin treatment, TFE3 was dephosphorylated and it was transported to the nucleus. The signaling pathway resulting in dephosphoylation of TFE3 remains to be determined. Nuclear translocation of TFE3 and transcription of GASE-containing genes was also activated when proteoglycan synthesis was inhibited or the CMP-sialic acid transporter was depleted (Taniguchi et al., 2015), suggesting that TFE3 regulation of Golgi homeostasis involves glycosylation. It will be interesting to see if this pathway is activated by increased flux of cargo during differentiation of secretory cells or trafficking of large cargo, or if different regions of the Golgi activate different stress response pathways, as recently proposed (Sasaki and Yoshida, 2015).

Another signaling pathway recently identified that may impact Golgi stress due to pathogen infection involves ADP-ribosylation factor (ARF)-4 and the transcription factor CREB3 (Reiling et al., 2013). Originally identified through a screen for brefeldin A-resistance, ARF4 levels increase when cells are exposed to Golgi disrupting treatments, and this requires the CREB3 transcription factor. Cells depleted of ARF4 or CREB3 contain increased levels of ARF1, 3, and 5, and are more resistant to infection with Chlamydia and Shigella. These results suggest that ARF4 may be induced by infection with these pathogenic bacteria to promote Golgi structural rearrangements required for efficient growth. How Golgi perturbation activates CREB3 is unknown. An earlier finding of caspase-2 involvement in infection with pathogenic bacteria is also intriguing in this light (Jesenberger et al., 2000).

## Golgi disruption in neurodegenerative diseases

Golgi fragmentation has been observed for decades in neurons from patients and in animal models of neurodegenerative diseases, including amyotrophic lateral sclerosis, Alzheimer's disease, Creutzfeld-Jacob disease and spinocerebellar ataxia type 2 (Gonatas et al., 2006). But does the fragmentation induce neuron dysfunction or is it simply a downsteam effect? Most evidence suggests that Golgi disruption occurs prior to cell death or disease phenotypes. In cortical neurons undergoing excitotoxicity or oxidative or nitrosyl stress, Golgi fragmentation precedes cell death, and both fragmentation and death could be blocked when Golgi structure was rescued with expression of a C-terminal GRASP65 fragment (Nakagomi et al., 2008). The C-terminal GRASP65 fragment allows formation of GRASP oligomers but cannot be phosphorylated, which is required for disassembly. Golgi fragmentation usually results in decreased or blocked trafficking to the cell surface. This would be expected to impair cellular function and could contribute to apoptosis.

In cell and animal models of Alzheimer's disease, increased Aβ processing from the amyloid precursor protein (APP) leads to Golgi fragmentation before cell death (Gonatas et al., 2006). A recent study shows that phosphorylation of GRASP65 by Cdk5 activated by Aβ (possibly through calcium signaling) results in reversible disassembly of the Golgi complex (Joshi et al., 2014). By contrast to other situations where Golgi fragmentation results in decreased cargo trafficking, the Wang group has shown that Aβ-induced fragmentation actually increases cargo trafficking. This results either directly or indirectly in positive feedback, where production of Aβ is increased and thus increases Golgi fragmentation. However, increased Aβ processing may block further accumulation of Aβ. It was recently shown that the intracellular domain of APP (released after Aβ processing and translocated to the nucleus) leads to reduced levels of machinery required for APP trafficking out of the Golgi, and thus reduced production of Aβ (Ceglia et al., 2015). Clearly, there is still much to learn about the role of the Golgi complex in Alzheimer's disease.

ACBD3 (also known as GCP60) is upregulated in cell culture models expressing the huntingtin (Htt) protein with expanded polyglutamine repeats, as well as in the brains of mice that model Huntington disease and in the striatum of Huntington's patients (Sbodio et al., 2013). ACBD3 is a ubiquitous peripheral Golgi protein that interacts with golgins and may regulate trafficking of fragments of golgin-160 generated by caspase cleavage (Sbodio et al., 2006). Interestingly, as mentioned above, ACBD3 is one of the genes shown to be upregulated by Golgi stress induced by monensin (Oku et al., 2011), which requires TFE3 (Taniguchi et al., 2015). In striatal neurons, increased levels of ACBD3 may lead to increased neurotoxicity due to its interaction with both Rhes and Htt. Rhes is a small G protein that is associated with Htt pathogenicity. Rhes is specifically expressed in the striatum (unlike Htt, which is widely expressed) and is thus thought to limit the site of degeneration in Huntington's disease to this region of the brain. Caspase-2 cleavage of Htt has also been implicated in pathology (Hermel et al., 2004). It will be interesting to see if any other binding partners of ACBD3 and caspase-2 cleavage at the Golgi are involved in Huntington's pathology.

## Outlook

Elucidation of the types of stresses that originate at the Golgi complex as well as the resulting signaling pathways will be required for a complete understanding of the role of this important organelle in neurodegeneration as well as other diseases. The identification of transcription factors that respond to Golgi stress, such as TFE3 and CREB3, will greatly aid the dissection of the signaling pathways. Determining how perturbation of Golgi structure and cleavage fragments of Golgi structural proteins influence gene expression is crucial. Finally, the consequences of Golgi fragmentation on cell homeostasis, including membrane traffic, need to be assessed for each type of Golgi stress uncovered.

### Conflict of interest statement

The author declares that the research was conducted in the absence of any commercial or financial relationships that could be construed as a potential conflict of interest.

## References

[B1] Boatright K. M. Salvesen G. S. (2003). Mechanisms of caspase activation. Curr. Opin. Cell Biol. 15, 725–731. 10.1016/j.ceb.2003.10.009 14644197

[B2] Bundis F. Neagoe I. Schwappach B. Steinmeyer K. (2006). Involvement of Golgin-160 in cell surface transport of renal ROMK channel: co-expression of Golgin-160 increases ROMK currents. Cell. Physiol. Biochem. 17, 1–12. 10.1159/000091454 16543716

[B3] Ceglia I. Reitz C. Gresack J. Ahn J. H. Bustos V. Bleck M.. (2015). APP intracellular domain-WAVE1 pathway reduces amyloid-beta production. Nat. Med. 21, 1054–1059. 10.1038/nm.3924 26280122 PMC4560977

[B4] Chiu R. Novikov L. Mukherjee S. Shields D. (2002). A caspase cleavage fragment of p115 induces fragmentation of the Golgi apparatus and apoptosis. J. Cell Biol. 159, 637–648. 10.1083/jcb.200208013 12438416 PMC2173109

[B5] Dippold H. C. Ng M. M. Farber-Katz S. E. Lee S. K. Kerr M. L. Peterman M. C.. (2009). GOLPH3 bridges phosphatidylinositol-4- phosphate and actomyosin to stretch and shape the Golgi to promote budding. Cell 139, 337–351. 10.1016/j.cell.2009.07.052 19837035 PMC2779841

[B6] Farber-Katz S. E. Dippold H. C. Buschman M. D. Peterman M. C. Xing M. Noakes C. J.. (2014). DNA damage triggers Golgi dispersal via DNA-PK and GOLPH3. Cell 156, 413–427. 10.1016/j.cell.2013.12.023 24485452 PMC4018323

[B7] Fava L. L. Bock F. J. Geley S. Villunger A. (2012). Caspase-2 at a glance. J. Cell Sci. 125, 5911–5915. 10.1242/jcs.115105 23447670

[B8] Fox R. M. Andrew D. J. (2015). Transcriptional regulation of secretory capacity by bZip transcription factors. Front. Biol. (Beijing). 10, 28–51. 10.1007/s11515-014-1338-7 25821458 PMC4374484

[B9] Gonatas N. K. Stieber A. Gonatas J. O. (2006). Fragmentation of the Golgi apparatus in neurodegenerative diseases and cell death. J. Neurol. Sci. 246, 21–30. 10.1016/j.jns.2006.01.019 16545397

[B10] Hermel E. Gafni J. Propp S. S. Leavitt B. R. Wellington C. L. Young J. E.. (2004). Specific caspase interactions and amplification are involved in selective neuronal vulnerability in Huntington's disease. Cell Death Differ. 11, 424–438. 10.1038/sj.cdd.4401358 14713958

[B11] Hicks S. W. Horn T. A. McCaffery J. M. Zuckerman D. M. Machamer C. E. (2006). Golgin-160 promotes cell surface expression of the Beta-1 adrenergic receptor. Traffic 7, 1666–1677. 10.1111/j.1600-0854.2006.00504.x 17118120

[B12] Hicks S. W. Machamer C. E. (2002). The NH2-terminal domain of Golgin-160 contains both Golgi and nuclear targeting information. J. Biol. Chem. 277, 35833–35839. 10.1074/jbc.M206280200 12130652

[B13] Hicks S. W. Machamer C. E. (2005). Golgi structure in stress sensing and apoptosis. Biochim. Biophys. Acta 1744, 406–414. 10.1016/j.bbamcr.2005.03.002 15979510

[B14] How P. C. Shields D. (2011). Tethering function of the caspase cleavage fragment of Golgi protein p115 promotes apoptosis via a p53-dependent pathway. J. Biol. Chem. 286, 8565–8576. 10.1074/jbc.M110.175174 21147777 PMC3048739

[B15] Jesenberger V. Procyk K. J. Yuan J. Reipert S. Baccarini M. (2000). Salmonella-induced caspase-2 activation in macrophages: a novel mechanism in pathogen-mediated apoptosis. J. Exp. Med. 192, 1035–1046. 10.1084/jem.192.7.1035 11015444 PMC2193309

[B16] Jiang Z. Hu Z. Zeng L. Lu W. Zhang H. Li T.. (2011). The role of the Golgi apparatus in oxidative stress: is this organelle less significant than mitochondria? Free Radic. Biol. Med. 50, 907–917. 10.1016/j.freeradbiomed.2011.01.011 21241794

[B17] Joshi G. Chi Y. Huang Z. Wang Y. (2014). Abeta-induced Golgi fragmentation in Alzheimer's disease enhances Abeta production. Proc. Natl. Acad. Sci. U.S.A. 111, E1230–E1239. 10.1073/pnas.1320192111 24639524 PMC3977293

[B18] Klumperman J. (2011). Architecture of the mammalian Golgi. Cold Spring Harb. Perspect. Biol. 3:a005181. 10.1101/cshperspect.a005181 21502307 PMC3119909

[B19] Lowe M. Lane J. D. Woodman P. G. Allan V. J. (2004). Caspase-mediated cleavage of syntaxin 5 and giantin accompanies inhibition of secretory traffic during apoptosis. J. Cell Sci. 117, 1139–1150. 10.1242/jcs.00950 14970262

[B20] Maag R. S. Mancini M. Rosen A. Machamer C. E. (2005). Caspase-resistant Golgin-160 disrupts apoptosis induced by secretory pathway stress and ligation of death receptors. Mol. Biol. Cell 16, 3019–3027. 10.1091/mbc.E04-11-0971 15829563 PMC1142444

[B21] Machamer C. E. (2013). Accommodation of large cargo within Golgi cisternae. Histochem. Cell Biol. 140, 261–269. 10.1007/s00418-013-1120-y 23821163 PMC3756474

[B22] Mala J. G. Rose C. (2010). Interactions of heat shock protein 47 with collagen and the stress response: an unconventional chaperone model? Life Sci. 87, 579–586. 10.1016/j.lfs.2010.09.024 20888348

[B23] Mancini M. Machamer C. E. Roy S. Nicholson D. W. Thornberry N. A. Casciola-Rosen L. A.. (2000). Caspase-2 is localized at the Golgi complex and cleaves golgin-160 during apoptosis. J. Cell Biol. 149, 603–612. 10.1083/jcb.149.3.603 10791974 PMC2174848

[B24] Miyata S. Mizuno T. Koyama Y. Katayama T. Tohyama M. (2013). The endoplasmic reticulum-resident chaperone heat shock protein 47 protects the Golgi apparatus from the effects of O-glycosylation inhibition. PLoS ONE 8:e69732. 10.1371/journal.pone.0069732 23922785 PMC3726774

[B25] Munro S. (2011). The golgin coiled-coil proteins of the Golgi apparatus. Cold Spring Harb. Perspect. Biol. 3:a005256. 10.1101/cshperspect.a005256 21436057 PMC3098672

[B26] Nakagomi S. Barsoum M. J. Bossy-Wetzel E. Sütterlin C. Malhotra V. Lipton S. A. (2008). A Golgi fragmentation pathway in neurodegeneration. Neurobiol. Dis. 29, 221–231. 10.1016/j.nbd.2007.08.015 17964175 PMC2261378

[B27] Oku M. Tanakura S. Uemura A. Sohda M. Misumi Y. Taniguchi M.. (2011). Novel cis-acting element GASE regulates transcriptional induction by the Golgi stress response. Cell Struct. Funct. 36, 1–12. 10.1247/csf.10014 21150128

[B28] Olsson M. Forsberg J. Zhivotovsky B. (2015). Caspase-2: the reinvented enzyme. Oncogene 34, 1877–1882. 10.1038/onc.2014.139 24882576

[B29] Ramirez I. B. Lowe M. (2009). Golgins and GRASPs: holding the Golgi together. Semin. Cell Dev. Biol. 20, 770–779. 10.1016/j.semcdb.2009.03.011 19508854

[B30] Reiling J. H. Olive A. J. Sanyal S. Carette J. E. Brummelkamp T. R. Ploegh H. L.. (2013). A CREB3-ARF4 signalling pathway mediates the response to Golgi stress and susceptibility to pathogens. Nat. Cell Biol. 15, 1473–1485. 10.1038/ncb2865 24185178 PMC3965854

[B31] Sasaki K. Yoshida H. (2015). Organelle autoregulation-stress responses in the ER, Golgi, mitochondria and lysosome. J. Biochem. 157, 185–195. 10.1093/jb/mvv010 25657091

[B32] Sbodio J. I. Hicks S. W. Simon D. Machamer C. E. (2006). GCP60 preferentially interacts with a caspase-generated golgin-160 fragment. J. Biol. Chem. 281, 27924–27931. 10.1074/jbc.M603276200 16870622

[B33] Sbodio J. I. Paul B. D. Machamer C. E. Snyder S. H. (2013). Golgi protein ACBD3 mediates neurotoxicity associated with Huntington's Disease. Cell Rep. 4, 890–897. 10.1016/j.celrep.2013.08.001 24012756 PMC3801179

[B34] Shi M. Vivian C. J. Lee K. J. Ge C. Morotomi-Yano K. Manzl C.. (2009). DNA-PKcs-PIDDosome: a nuclear caspase-2-activating complex with role in G2/M checkpoint maintenance. Cell 136, 508–520. 10.1016/j.cell.2008.12.021 19203584 PMC5647584

[B35] Taniguchi M. Nadanaka S. Tanakura S. Sawaguchi S. Midori S. Kawai Y.. (2015). TFE3 is a bHLH-ZIP-type transcription factor that regulates the mammalian Golgi stress response. Cell Struct. Funct. 40, 13–30. 10.1247/csf.14015 25399611

[B36] Walter P. Ron D. (2011). The unfolded protein response: from stress pathway to homeostatic regulation. Science 334, 1081–1086. 10.1126/science.1209038 22116877

[B37] Wang Y. Seemann J. (2011). Golgi biogenesis. Cold Spring Harb. Perspect. Biol. 3:a005330. 10.1101/cshperspect.a005330 21690214 PMC3179335

[B38] Williams D. Hicks S. W. Machamer C. E. Pessin J. E. (2006). Golgin-160 Is Required for the Golgi Membrane Sorting of the Insulin-responsive Glucose Transporter GLUT4 in Adipocytes. Mol. Biol. Cell 17, 5346–5355. 10.1091/mbc.E06-05-0386 17050738 PMC1679696

[B39] Xiang Y. Wang Y. (2010). GRASP55 and GRASP65 play complementary and essential roles in Golgi cisternal stacking. J. Cell Biol. 188, 237–251. 10.1083/jcb.200907132 20083603 PMC2812519

